# An excitatory GABA loop operating *in vivo*

**DOI:** 10.3389/fncel.2015.00275

**Published:** 2015-07-16

**Authors:** Guadalupe Astorga, Jin Bao, Alain Marty, George J. Augustine, Romain Franconville, Abdelali Jalil, Jonathan Bradley, Isabel Llano

**Affiliations:** ^1^Laboratory of Cerebral Physiology, CNRS and University Paris DescartesParis, France; ^2^Lee Kong Chian School of Medicine, Nanyang Technological UniversitySingapore, Singapore; ^3^Institute of Molecular and Cell BiologySingapore, Singapore; ^4^Center for Functional Connectomics, Korea Institute of Science and TechnologySeoul, South Korea

**Keywords:** calcium, GABA, cerebellum, parallel fibers, interneurons

## Abstract

While it has been proposed that the conventional inhibitory neurotransmitter GABA can be excitatory in the mammalian brain, much remains to be learned concerning the circumstances and the cellular mechanisms governing potential excitatory GABA action. Using a combination of optogenetics and two-photon calcium imaging *in vivo*, we find that activation of chloride-permeable GABA_A_ receptors in parallel fibers (PFs) of the cerebellar molecular layer of adult mice causes parallel fiber excitation. Stimulation of PFs at submaximal stimulus intensities leads to GABA release from molecular layer interneurons (MLIs), thus creating a positive feedback loop that enhances excitation near the center of an activated PF bundle. Our results imply that elevated chloride concentration can occur in specific intracellular compartments of mature mammalian neurons and suggest an excitatory role for GABA_A_ receptors in the cerebellar cortex of adult mice.

## Introduction

In recent years, potent effects of presynaptic axonal GABA_A_ receptors (GABA_A_Rs) have been described in various regions of the mammalian brain (Trigo et al., 2008). Paradoxically, although GABA is known to be the canonical inhibitory neurotransmitter at mammalian central synapses, activation of presynaptic GABA_A_Rs often results in excitation. For example, in cerebellar slices, GABA spillover during molecular layer interneuron (MLI) activity leads to excitation of parallel fibers (PFs; Stell et al., 2007; Pugh and Jahr, 2011; Stell, 2011; Dellal et al., 2012). Because MLIs are excited by glutamate released by PFs, this raises the possibility of a positive feedback “GABA loop” where the excitability of a PF beam is enhanced by PFs exciting MLIs. Whereas the functioning of the various elements of this potential loop have been demonstrated separately in slices, it remains unclear whether the entire loop actually functions as a positive gain control for the PF-MLI circuit. Providing evidence for the existence and functional relevance of this loop *in vivo* is one of the major goals of this work.

The ability of GABA to increase PF excitability has been demonstrated by application of GABA_A_R agonists to GABA_A_Rs located in PFs (Stell et al., 2007; Pugh and Jahr, 2011; Stell, 2011; Dellal et al., 2012). The mechanisms behind this enhanced PF excitability remain unclear. One intriguing possibility is that the intracellular Cl^−^ concentration (Cl_i_) of PFs is elevated, such that activation of GABA_A_Rs leads to Cl^−^ efflux and depolarization. Although evidence from slices from various brain regions indicates high Cl_i_ levels in axons (Price and Trussell, 2006; Szabadics et al., 2006) which can lead to excitatory GABA effects (see Trigo et al., 2008, for review), it remains to be determined whether the same occurs in adult rodents *in vivo*. Moreover, several reports suggest that Cl^−^ homeostasis may be different in brain slices vs. the intact brain and results in high Cl_i_ in slices (Bregestovski and Bernard, 2012; Dzhala et al., 2012; Glykys et al., 2014) thus questioning the general relevance of excitatory effects recorded in slices. In particular severing of axon branches during slice preparation may lead to artificially elevated axonal Cl_i_ levels. Independently of the mode of action of axonal GABA_A_Rs, it is unclear whether the GABA released by MLI activation *in vivo* can diffuse far enough to stimulate GABA_A_Rs located in PFs. Nevertheless, a recent electrophysiological and imaging study of the cerebellar cortex in awake mice shows robust firing rate increases in individual MLIs as well as Ca_i_ rises in large MLI clusters during locomotion. The increases in MLI activity are sustained for several seconds, as long as the motor activity lasts (Ozden et al., 2012). This raises the possibility that spillover of GABA occurs during behavioral episodes.

We therefore searched for excitatory effects mediated by axonal GABA_A_Rs *in vivo*, under experimental conditions that preserve brain tissue integrity and physiological Cl^−^ homeostasis. We used an anesthetized mouse preparation which allows for the recording of stable signals and is amenable to pharmacological manipulation, an essential tool for the present study. Our results provide support for the existence and functional relevance of a GABAergic positive feedback loop in the molecular layer of the cerebellum. More generally, they suggest that GABA can have an excitatory action *in vivo*.

## Materials and Methods

Experimental procedures complied with the animal care guidelines of the host institution and were approved by the “Prefecture de Police” in agreement with the European Directive 86/609/EEC and by the ethical committee of the University Paris Descartes.

### GCaMP Expression in the Cerebellar Cortex

Four to five week old mice heterozygous for an allele driving CRE recombinase under the control of the parvalbumin (PV) promoter (Hippenmeyer et al., 2005), *PV-Cre* mice, were deeply anesthetized with intraperitoneal injection of ketamine (14.8 μg/g) and xylazine (20 μg/g) and mounted in a stereotaxic frame. A midline sagittal incision exposed the cranium over the cerebellar vermis. At the site of injection (6 mm from Bregma and 0.6 mm lateral) a 0.5 mm burr hole was drilled and a 34-gauge stainless steel beveled needle (WPI-nanofil) was slowly descended 0.4 mm through a slit cut in the meninges. After a 2 min pause, the needle was retracted 50 μm and then 1.2 μl of AAV2/1.hSynap.Flex.GCaMP3.WPRE.SV40 (University of Pennsylvania Vector Core, Philadelphia, PA, USA) were injected at a rate of 0.1 μl/min. Once completed, the needle was left in place an additional 10 min before being withdrawn, the scalp sutured, and the mouse kept under a warming lamp until it recovered from the anesthesia before finally being returned to standard housing.

Although PV is expressed both in Purkinje cells (PCs) and in MLIs, in the present expression system PCs did not express the GCaMP protein (Figure 7A). This may reflect the differential activity of the hSyn promoter, and has been described for a different genetically-encoded Ca^2+^ indicator (Kuhn et al., 2012). The specificity of MLI expression was confirmed in 26 animals by confocal imaging of slices prepared from mice used for *in vivo* imaging. In a subset of these experiments immunocytochemistry was performed, as detailed below.

### GCaMP Imaging *in vivo*

Three to seven weeks after stereotaxic vector delivery, mice were prepared for imaging as described in detail previously (Franconville et al., 2011). The time of expression was chosen to avoid GCaMP over expression, phenotypically determined by GCaMP signal being excluded from the nucleus (Tian et al., 2009). During imaging anesthesia was delivered via an intraperitoneal catheter and vital parameters were continuously monitored using a Pulse Oxymeter system (Starr Life Sciences, PA, USA). The craniotomy was bathed in 1–2 ml of Hepes-buffered saline (HBS) of composition (in mM): 145 NaCl, 2.5 KCl, 2 CaCl_2_, 1 MgCl_2_, 5 glucose, 10 HEPES-Na, set to pH 7.4, pre-warmed to 37°C, replaced every 15–20 min. Two-photon laser scanning imaging was performed with a custom-built set-up (details in Franconville et al., 2011). Excitation wavelength for Ca^2+^-dependent GCaMP3 signals was 910 nm. In some of the pharmacological experiments, sequences of 910 and 810 nm excitations were performed in control period and following drug application and ratios of the emitted fluorescence were calculated (F_910_/F_810_). GCaMP3 fluorescence when excited at 810 nm is not Ca^2+^-dependent and therefore this protocol allowed us to control for changes in fluorescence unrelated to Ca_i_ changes.

### Extracellular Stimulation *in vivo*

Theta-glass pipettes (tip diameter 1–2 μm) filled with HBS and 20 μM Alexa 594 dye to aid visualization, were introduced 30–50 μm into the molecular layer. The surface of the brain was visualized with a CCD camera under reflected light using μManager software (Edelstein et al., 2010). Imaging was done 40–60 μm deeper than the pipette tip and at a lateral distance of more than 130–180 μm from the site under study, to avoid direct stimulation of the neurons in that field. Ag-AgCl electrodes connected the pipette to an isolated pulse stimulator (AM-systems, Sequim, WA, USA) which delivered 100–200 μs long pulses at −30 to −80 V amplitude.

### Muscimol Iontophoresis *in vivo*

Borosilicate glass pipettes (tip diameter approximately 1 μm) were filled with 50 mM muscimol (Abcam, Cambridge, UK) diluted in a citric acid buffer (pH:3.7) and 20 μM of Alexa 594 dye. They were connected by Pt electrodes to an iontophoretic drug ejection system (MVCSC, npi electronic, Tam, Germany). A retaining current of −20 nA was applied to avoid leak of muscimol from the pipette; muscimol was ejected by current pulses of 100 nA applied during 1–2 s. Based on previous diffusion measurements in the cerebellar cortex (Nicholson and Phillips, 1981; Rice et al., 1993) we estimated that the muscimol concentration released from the tip of the pipette in the present experiments ranged from 50 to 100 μM for a radius of 90 μm.

### Pharmacological Protocols and Chemical Reagents

When assessing drug effects on the signals elicited by PF stimulation or by muscimol iontophoresis *in vivo*, stimulations were delivered at 4–5 min intervals with a minimum of five repetitions before adding the drug. Drug(s) were added to the bath above the craniotomy and stimulation continued at the same rhythm after drug application. Tetrodotoxin, SR95531, GYKI 53655,5,7-dichlorokynurenic acid (DCK) and CPCCOEt were purchased from Tocris (Bristol, UK) or Abcam.

### Photostimulation of channelrhodopsin2 (ChR2)

Mice expressing channelrhodopsin2 (ChR2) under the control of the neuronal nitric oxide synthase (nNOS) promoter (Kim et al., 2014) were used to achieve specific photostimulation of MLIs. In a first series of experiments, cerebellar slices from mice aged PN 37–44 were used to develop photostimulation protocols. Sagittal slices were prepared using a low Na^+^ saline designed for slicing of adult mice brain (Zhao et al., 2011) having the following composition (in mM): 93 N-methyl-D-glucamine, 2.5 KCl, 1.25 NaH_2_PO_4_, 25 NaHCO_3_, 0.5 CaCl_2_, 10 MgCl_2_, 20 Hepes, 5 Na ascorbate, 2 thiourea, 3 Na pyruvate and 25 glucose (HCl added to bring pH to 7.4). Slices were maintained for 30–40 min at 34°C in standard recording saline (in mM: 125 NaCl, 2.5 KCl, 1.25 NaH_2_PO_4_, 25 NaHCO_3_, 2 CaCl_2_, 1 MgCl_2_, and 10 glucose) prior to transfer to the recording set-up at 34°C. Two-photon imaging was performed to visualize YFP fluorescence using an excitation wavelength of 910 nm. Loose-seal recordings from MLI somata were performed as detailed elsewhere (Franconville et al., 2011). Photostimulation at 470 nm was delivered by an LED-based system (OptoFlash, Cairn Research, Kent, UK) whose output was coupled to a 1 mm optic fiber mounted on a manipulator allowing positioning inside the solution bathing the slice.

*In vivo* experiments were done with the same mouse line at PN32 to PN168. Protocols for anesthesia, imaging and PF stimulation were as for the imaging experiments. Extracellular recordings were made with an Axopatch 200 A amplifier (Molecular Devices, Sunnyvale, CA, USA) set to the I-Clamp configuration. Borosilicate glass pipettes were filled with HBS containing 20 μM Alexa 594. For analyzing the effects of photostimulation on spike activity, pipettes (resistances 10–20 MΩ) were introduced in the molecular layer until a stable unit recording was identified. For studying the effects of photostimulation of the PF volley, recording pipettes (resistances ranging from 5 to 10 MΩ) were placed at the same Z plane and 400–600 μm laterally with respect to the stimulating electrode. The recording pipette was moved diagonally (20–50 μm) until the amplitude of the volley was optimal. To avoid saturating levels of stimulation, the voltage was then reduced until reproducible signals could be recorded after each stimulation. The protocol for volley experiments consisted of delivering a set of 6 extracellular stimulations at 1 Hz; the LED was turned on during the second and third pulses. This protocol was repeated 20 times.

The same photostimulation system coupled to a 1 mm fiber was used for experiments *in vitro* and *in vivo*. The power out of the fiber was set at 6 mW for the *in vitro* experiments and at 8 mW for the *in vivo* experiments.

### Two-Photon Data Analysis

Two-photon imaging data were analyzed with custom-written routines either in R environment or in Igor Pro (Wavemetrics, Lake Oswego, OR, USA). Background fluorescence was estimated as the fluorescence value averaged over the 20–30 pixels with the lowest value across all the frames of the sequence. Automatic region of interest (ROI) detection and estimates of fluorescence changes were performed as detailed before (Franconville et al., 2011). Regions of interest (ROIs) were detected on reference images. Two types of reference images were used: either images of the average intensity over time, when ROIs detection based on morphology was desired, or local correlation images (neighborhood correlation map; Junek et al., 2009) when detection based on activity was used. Local correlation images are formed by computing the correlation (in time) of each pixel with its direct neighbors. We used the square of the correlation for detection, as it empirically yielded more defined ROIs. References images were thresholded using k-means clustering with two levels on the pixel intensity values, and regions were determined by labeling the connected components of the thresholded result.

A ROI was considered as responding if the peak ΔF/Fo value was larger than 5%. For pharmacological analysis, only those experiments with at least five reproducible responses in the control period were accepted. Peak values for the ΔF/Fo signals were averaged over the control runs and over the last three runs in drug(s).

### Electrophysiological Data Analysis

Electrical extracellular recordings of unitary action potentials and of volley signals were analyzed in Igor Pro. The Spike detection option of Neuromatic software was used to detect unitary spikes in PCs and MLIs. For analysis of the volley signal, which consisted of two major negative waves, named N_1_ and N_2_ in accord with previous reports in anesthetized mice (Wang et al., 2011), times to N_1_ for individual traces were analyzed with a peak detection routine.

### Statistical Analysis

Values for pooled data are given as mean ± SEM. All statistical comparisons were made with paired Student’s *t*-test and *p* values are reported for each comparison. The Pearson’s coefficient (Pr) was used to assess the significance of correlations.

#### Immunocytochemistry

At the end of four *in vivo* experiments, the mice were decapitated while still under anesthesia, the cerebellum was immediately removed and fixed overnight in PBS containing four percent of paraformaldehyde. Fifty micrometer thick transverse slices were prepared using a vibrating slicer (Leica VT 1000S, Leica Biosystems, Nussloch, Germany). The slices were first incubated in PBS containing 0.3% Triton and 10% fetal bovine serum for 5 h at room temperature then at 4°C overnight with guinea pig polyclonal serum anti calbindin D28k (Synaptic Systems, Göttingen, Germany) diluted to 1:1000 in PBS containing 1 mg/ml of Bovine Serum Albumine (BSA). After three washes in PBS, the slices were incubated for 3 h with secondary goat antibodies anti-guinea pig IgG conjugated to Alexa fluor 647 (1/500, Molecular Probes, Eugene, OR, USA). Slices were mounted between slides and cover slip with Vectashield Mounting Medium (Vector lab, Burlingame, CA, USA). Images were acquired using an LSM 510 confocal microscope (Zeiss, Jena, Germany) equipped with Plan-Neofluar 40×/1.3 oil objective and appropriate laser excitation (488 and 633 nm) and emission filters (BP 505–530 and LP 650). Image analysis and projections were done with LSM and ImageJ Software.

## Results

### Inhibitory Effects of GABA_A_R Blockade

**Figure 1:**
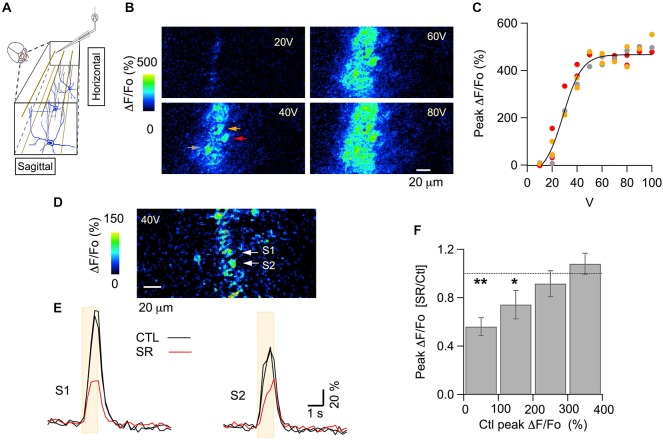
**GABA_A_Rs modulate PF-evoked somatic Ca_i_ rises in MLIs *in vivo*. (A)** Scheme for the electrical stimulation protocol used in the anesthetized mouse. Parallel fibers (PFs) are depicted in brown, MLIs in blue. Pulses were delivered through a theta-glass pipette placed 30–50 μm above the imaged horizontal plane and ~150 μm lateral to this site, to avoid direct stimulation of the neurons in that field. **(B)** ΔF/Fo images from two-photon laser scanning of MLIs expressing GCaMP3. Each panel shows the peak of the response to electrical stimulation (100 pulses of 100 μs duration delivered at 100 Hz) at the indicated intensity. **(C)** Stimulus-response curve for the peak ΔF/Fo from the three molecular layer interneuron (MLI) somata indicated by color arrows in **(B)**. The solid line corresponds to the fit of the data by a sigmoidal function, yielding 514 ± 67% for the maximum value and 29 ± 3 V for the abscissa at half maximum. **(D,E)** A different example for the signals elicited by 40 V stimulus trains. **(D)** ΔF/Fo image at the peak of the response. Two MLI somata (S1 and S2) are identified by white arrows. **(E)** Time course for the Ca_i_ rises in the two MLI somata indicated by white arrows in **(D)** in control saline (black traces) and 35–50 min after addition of the GABA_A_R specific blocker SR95531 (75 μM) to the solution bathing the craniotomy (red trace). Each trace is the average of three consecutive runs. To illustrate the signal stability prior to drug application, control runs were averaged over two control periods. The beige boxes denote the time of extracellular stimulation. **(F)** The effect of SR95531 depends on the initial amplitude of the PF-evoked signal. Data was pooled from 37 somata recorded from seven mice. Ratios of peak ΔF/Fo in control over drug were grouped by bins according to the peak ΔF/Fo value in the control runs. Values for each soma were averaged over three consecutive runs for the control period and for the same number of runs taken 30–50 min after drug application. Error bars are mean ± SEM. Number of cells and *p* value for paired Student’s *t*-test were: 9 and 0.002 for the first bin; 11 and 0.05 for the second bin; 9 and 0.47 for the third bin; 8 and 0.31 for the fourth bin. *denotes groups with *p* ≤ 0.05; **groups with *p* ≤ 0.01. In six of these experiments, comparison of the F_910_/F_810_ values (see “Materials and Methods” Section) in control and after addition of SR95531 yielded no significant difference (*p* value for paired Student’s *t*-test: 0.34) suggesting that no significant changes in basal Ca_i_ levels occurred.

A classical method to assess the polarity of GABA_A_R action is to compare neuronal activity before and after application of a GABA_A_R blocker; if such a blocker has an inhibitory effect, this indicates an excitatory effect of GABA_A_Rs in the signaling circuit (Ben-Ari et al., 1989). In adult brain tissue, GABA_A_Rs are usually inhibitory, and therefore GABA_A_R blockers are usually excitatory. Examples of such blocker-induced excitation in the cerebellar cortex can be found in imaging studies both *in vivo* and in slices. Thus, in anesthetized mice, bicuculline increases the activity-dependent flavoprotein autofluorescence signals elicited by stimulation of PFs (Gao et al., 2006). In slices, a recent two photon imaging study using Fura-2 AM loading showed that the amplitude of the Ca_i_ rises evoked in the somata of granule cells (GC) and PCs by trains of mossy fiber stimulation is larger in the presence of the specific GABA_A_R blocker SR95531 (Gandolfi et al., 2014). In order to investigate possible excitatory GABA_A_R actions in the cerebellar cortex *in vivo*, we examined the effects of SR95531 on PF-induced signals in MLIs, the GABAergic interneurons which are postsynaptic to PFs. In order to minimize invasive effects we optically monitored MLI calcium as an indicator of postsynaptic activation by expressing the fluorescent optogenetic Ca^2+^ indicator GCaMP3 (Tian et al., 2009). PFs were stimulated by applying repetitive pulses to a theta-glass pipette placed in the molecular layer. MLIs were imaged in a horizontal plane along the 20–40 μm wide beam which resulted from the stimulation (Figure 1A; see “Materials and Methods” Section). *In vivo*, GC respond to depolarizing current injection with increases in firing rate to frequencies of several 100 Hz and the responses show no accommodation (Chadderton et al., 2004), indicating that these neurons have the ability to sustain high frequency firing. Evidence that such sustained firing indeed occurs in behaving animals has come forward from imaging work in mice showing persisting enhanced activity throughout locomotion episodes lasting several 10 s of seconds (Ozden et al., 2012). In view of these studies we applied 1 s long trains of stimuli at 100 Hz. We observed responses that were graded with stimulus intensity in MLIs along the PF beam (Figures 1B,C). The saturation of the response apparent in Figure 1C likely reflects the fact that all PFs presynaptic to a given MLI are activated at the highest intensities, as well as the non-linear Ca^2+^-dependence of GCaMP3 (Tian et al., 2009).

The somatic MLI responses analyzed on the beam-center were examined at moderate stimulus intensity (40 V in the example shown in Figures 1D,E), corresponding to submaximal PF recruitment. These responses decreased upon application of SR95531 (75 μM), suggesting an excitatory action of GABA_A_Rs (Figures 1D,E). On the other hand, responses to larger intensities typically were not affected by SR95531 as quantified below. To compensate for differences in the position of the dose-response curve along the voltage axis among different experiments, responses were classified accordingly to the amplitude of the control response. Group data classified with respect to control peak ΔF/Fo amplitude from seven animals are displayed in Figure 1F. They show a significant reduction of the responses with control peak amplitudes in the ranges 0–100% and 100–200%, but no significant change for the 200–300% and 300–400% ranges. These results indicate, in agreement with data in slices (Stell, 2011) that the effect of SR95531 disappeared at saturating stimulation intensities. This suggests that GABA_A_Rs can modulate the number of activated PFs.

**Figure 2:**
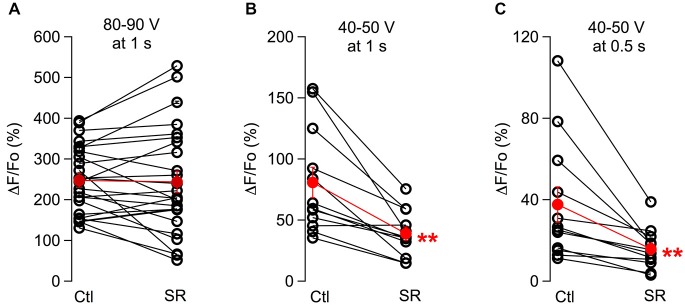
**The effect of GABA_A_Rs block depends on the stimulation protocol.** Pair plots for peak ΔF/Fo values in control conditions and in the presence of SR95531 are shown for responses to 100 Hz, 1 s long trains using maximal stimulus intensities **(A)** and low stimulus intensities **(B). (C)** Analysis of the same data set as in **(B)** at 0.5 s. For all cases, values for each soma were averaged over 3 consecutive runs for the control period and for the same number of runs taken 30–50 min after drug application. Open symbols represent individual somata and red dots the corresponding average; error bars are SEM. **p ≤ 0.01.

When comparing results from the two stimulus intensities used, we found that SR95531 significantly reduced responses to 40–50 V stimuli but failed to affect the responses to 80–90 V stimulus (Figures 2A,B; ratios of the peak response in drug over control were 0.52 ± 0.06; *n* = 12 somata from three animals, p: 0.001 for the 40–50 V stimulations and 0.99 ± 0.07, *n* = 25 somata from seven animals for the 80–90 V stimulation). These results are consistent with the results of Figure 1F indicating that SR95531 reduces submaximal responses but has no effect on maximal responses. Next we asked whether SR95531 would reduce responses to still weaker stimuli, in particular to shorter trains at 40–50 V. Even though only 1 s long trains were used during the experiments, we could infer the effects of SR95531 on shorter trains by examining the amounts of reduction at different time points during the train. In 12 somata from three animals, ΔF/Fo ratios in the drug over control were similar after 50 stimulations (at 500 ms: 0.48 ± 0.06; *p*: 0.006) and after 100 stimulations (at 1 s: 0.52 ± 0.06; *p*: 0.001), indicating that SR95531 reduced the overall response amplitude without modifying its time course, so that the extent of reduction was independent of stimulus duration (Figures 2B,C).

In summary, these results strongly indicate the existence of excitatory effects of GABA_A_Rs in the cerebellar molecular layer *in vivo*. In addition they suggest that GABA spillover from MLIs can activate GABA_A_Rs in PFs in the intact PF-MLI circuit, and that their activation controls PF firing.

### Excitatory Effects of GABA_A_R Activation

**Figure 3:**
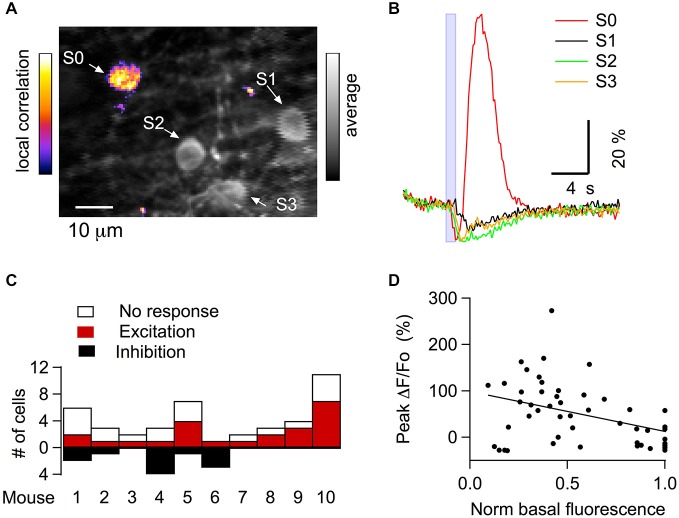
**Ca_i_ signals elicited in MLIs by iontophoretic application of the selective GABA_A_R agonist muscimol *in vivo*. (A)** Merged image of the average of 30 pre-stimulus two-photon laser scanning images (gray scale) and the correlation image (see “Materials and Methods” Section; fire pseudo color scale) for the same field following a muscimol challenge. Four MLI somata (S0, S1, S2 and S3) are identified by white arrows. **(B)** Individual MLI somata (identified by arrows in **A**) in the same visual field respond differently to a 1 s long muscimol iontophoresis (50 mM in the pipette), as indicated by the blue box. The estimated concentration of muscimol released is 50–100 μM (see “Materials and Methods” Section). The pipette was located approximately 20–50 μm above the imaged field. **(C)** Pooled data showing the distribution of the response type induced by muscimol iontophoresis across animals (10 mice). This analysis was performed only in those experiments in which more than two somata were present in the imaged field. **(D)** The excitatory effect of muscimol is negatively correlated to the somatic basal fluorescence level. For each field imaged, the pre-stimulus fluorescence levels for all somata were normalized to the value of the highest soma in the field (solid line: linear regression fit; regression coefficient = −0.41; *p* < 0.001; pooled data from 10 mice).

**Figure 4:**
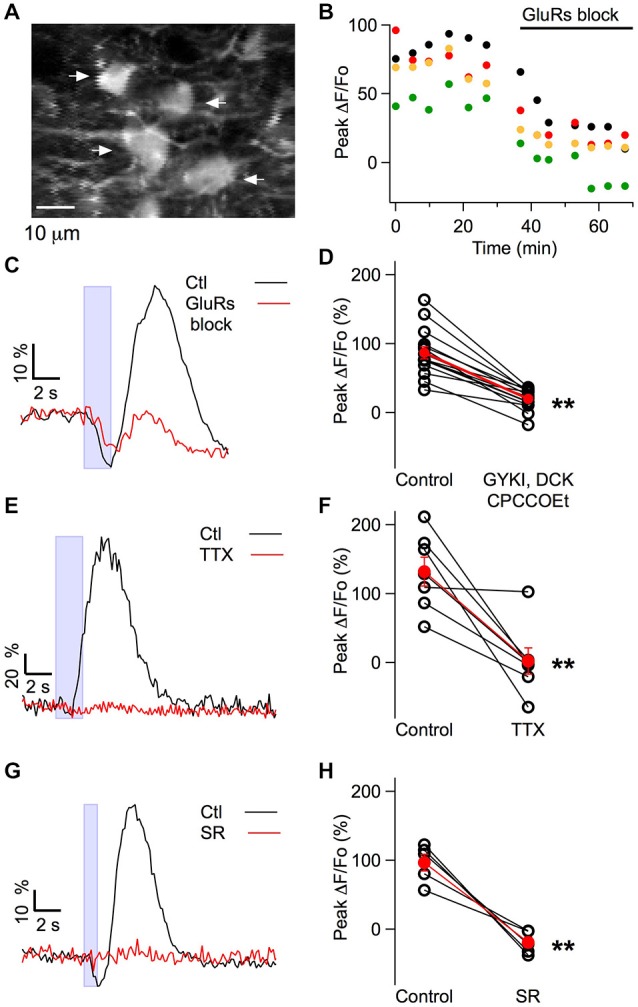
**Pharmacological profile of the muscimol-evoked Ca_i_ rises obtained in MLIs *in vivo*. (A)** Average of 30 pre-stimulus two-photon laser scanning images of MLIs. **(B)** A cocktail of glutamate receptor blockers (GYKI 100 μM, APV 20 μM, CPCCOEt 100 μM) decreased the peak somatic ΔF/Fo induced by muscimol in the four MLIs somata indicated by arrows in **(A). (C,E,G)** Representative examples for the time course of the somatic fluorescence changes induced by muscimol iontophoresis in control conditions and after addition of the indicated blockers to the solution bathing the craniotomy. Blue boxes denote the time of muscimol application. Concentrations for the glutamate receptor blockers in **(C,D)** were as in **(B)**; in **(E,F)** TTX was used at 4 μM; in **(G,H)** SR95531 was used at 75 μM. Note that in **(C)**, the fluorescence increase (reflecting excitation) was highly sensitive to the glutamate receptor antagonists whereas the decrease in fluorescence (reflecting inhibition) was unaffected. **(D,F,H)** Pair plots for the peak somatic ΔF/Fo values in control conditions and in the presence of the indicated drugs. Open symbols represent individual somata and red dots the corresponding average; error bars are SEM. The average block by the glutamate antagonists was 78 ± 5%, *n* = 15 somata from six mice (*p* = 3 × 10^−7^). TTX completely blocked the response with an average block of 98 ± 17%, *n* = 7 somata from six mice (*p* = 0.005). SR95531 completely blocked the response with an average block of 117 ± 6%, *n* = 5 somata from four mice (*p* = 0.004). All *p* values are for paired Student’s *t*-test. **p < 0.01. F_910_/F_810_ values (see “Materials and Methods” Section) were monitored in two of the glutamate blocker experiments in control and in the presence of the blockers and no significant difference was found (*p* value for paired Student’s *t*-test: 0.50) arguing against significant changes in basal Ca_i_ levels.

To investigate further the possible site of GABA-induced excitation we next performed experiments using direct GABA_A_R stimulation. In cerebellar slices, local application of GABA_A_R agonists in the molecular layer leads to the firing of PFs and subsequent activation of MLIs (Stell et al., 2007; Pugh and Jahr, 2011; Stell, 2011; Dellal et al., 2012). We examined whether similar effects occur *in vivo*, using iontophoresis to locally apply the GABA_A_R agonist, muscimol, in the molecular layer of mice expressing GCaMP3 while recording the activity of MLIs. The basal fluorescence level in individual MLIs was heterogeneous (Figure 3A), reflecting differences in the level of GCaMP3 expression and in the basal activity of individual cells (Franconville et al., 2011). The pattern of responses to muscimol recorded in MLIs varied depending on a number of factors. If the imaging plane was at the level of the tip of the iontophoretic pipette, only inhibition was observed suggesting that direct muscimol effects were inhibitory. However, if the plane was located 30–50 μm below the tip of the iontophoresis pipette, corresponding to the expected location of MLIs postsynaptic to the PFs directly exposed to muscimol, as in Figure 3B, mixed excitation and inhibition was observed. Another important factor was agonist concentration. Comparatively low muscimol concentrations (0.5 mM in the pipette, as in Franconville et al., 2011) produced inhibitory responses, indicated by a decrease in fluorescence and reflecting inhibition of action potentials (APs). Higher muscimol concentrations (50 mM in the pipette, yielding an estimated concentration of released muscimol of 50–100 μM; see “Materials and Methods” Section), produced approximately 50% of excitatory responses, indicated by an increase in fluorescence. From 53 MLIs in 10 animals measured under optimal conditions (located 30–50 μm below the tip of the iontophoresis pipette, and stimulated with 50 mM muscimol in the pipette), 23 were excited by muscimol application, 11 had a purely inhibitory response, and 19 did not respond (Figure 3C). The excitatory responses were often preceded by an inhibitory phase (examples in Figures 3, 4). Inhibitory and excitatory responses were often present in the same field of view (Figures 3A,B) arguing against the metabolic state of the animal being a factor. In such fields, somata displaying inhibitory and excitatory responses appeared to be randomly distributed, suggesting that response polarity was not simply determined by the muscimol concentration reached at each recorded somata. Moreover, we found that the probability of observing an excitatory response was higher in MLIs with lower basal fluorescence levels (Figure 3D; Pr: 0.41; *p* < 0.01). This correlation could arise from a dependence of the response on the Ca^2+^ buffering added by GCaMP3, although the high intrinsic Ca^2+^ buffering power of MLIs (Collin et al., 2005) argues against this interpretation. Another explanation may be that MLIs with low resting firing rate are more responsive to muscimol. Analysis of the MLIs exhibiting excitatory responses yielded an average somatic Ca_i_ rise with peak value of 104 ± 10% (*n* = 23 somata from 10 animals). Analysis of the surrounding neuropil in the molecular layer gave slightly larger responses (112 ± 7%, *n* = 13 from three animals). These results show that activation of GABA_A_Rs can cause excitation of MLIs *in vivo*.

### Pharmacological Profile of the Muscimol-Induced Excitation

We next investigated the mechanisms underlying the excitatory responses to iontophoresis of muscimol. Reports of somatodendritic Ca_i_ elevation by muscimol or GABA applications have mostly been restricted to neonatal preparations (e.g., Eilers et al., 2001), but MLIs are a rare exception (Chavas et al., 2004), so that it could not be excluded that the Ca_i_ elevation was elicited by GABA_A_Rs located on MLIs. Alternatively, the Ca_i_ elevation could be a consequence of muscimol-induced activation of GABA_A_Rs in PFs, as described earlier. If the former, muscimol-induced responses should be insensitive to blockers of APs and of glutamate receptors, whereas if the latter, they should be sensitive to these blockers (Chavas et al., 2004; Stell et al., 2007). To test this, we applied a cocktail of glutamate receptor blockers and found that this dramatically reduced the response (mean reduction in amplitude: 78 ± 5%, 15 MLIs from six animals; *p* = 3 × 10^−7^; Figures 4A–D). Neuropil signals in the molecular layer were similarly decreased (mean reduction in amplitude: 73 ± 5%, 13 locations in three animals; *p* = 1 × 10^−9^). Likewise, application of tetrodotoxin abolished the response in all except 1 experiment (mean reduction: 99 ± 17%, seven MLIs from six animals including the non-responding experiment; *p* = 0.005; Figures 4E,F). The early inhibition observed during biphasic responses was not affected by the glutamate blockers (mean ratio of the −ΔF/Fo in drug to control: 1.06 ± 0.04, four MLI from four animals). These results strongly suggest that while inhibitory signals arise from direct inhibition of MLIs, the excitatory responses are due to an indirect mechanism involving PF activation and subsequent glutamate release.

To exclude the possibility that the iontophoretic current applied to the muscimol-containing pipette directly depolarized MLIs or PFs, we performed additional experiments with SR95531. This drug eliminated the muscimol-induced excitation (mean reduction: 117 ± 6%; five MLI in four animals; *p* = 0.004), ruling out the possibility of direct stimulation (Figures 4G,H).

Taken together, these results suggest that muscimol acts on PF GABA_A_Rs *in vivo*, resulting in an increase in PF firing rate and a subsequent activation of MLIs.

### Action of GABA_A_Rs on the PF Volley

**Figure 5:**
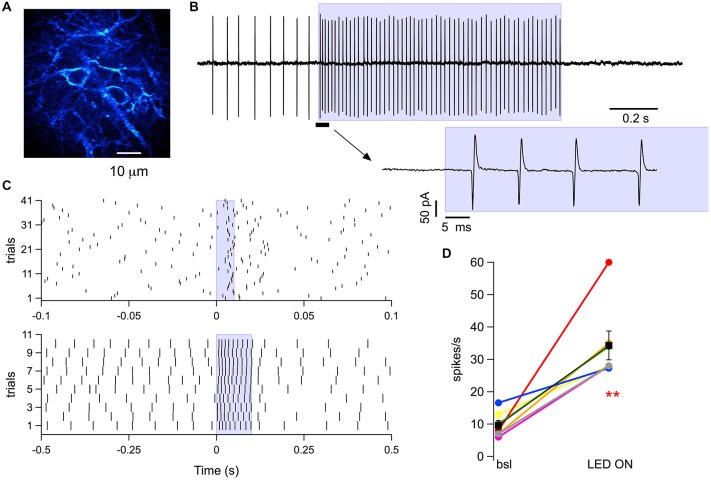
**MLI responses to photostimulation in cerebellar slices. (A)** Two-photon laser scanning image from a sagittal slice of a ChR2-YFP mouse, showing somata and neurites of MLIs (post-natal age 37). **(B)** A 1 s long wide-field photostimulation using a 470 nm LED coupled to a 1 mm optical fiber placed inside the slice chamber induced an increase in MLI spike frequency that persisted throughout the light pulse and was followed by a long pause. Spikes were recorded in the loose seal cell-attached configuration. The blue box denotes the time of photostimulation. The inset below displays, at an expanded time scale, the portion of the recording just before and during the onset of the light pulse (as indicated by the solid black bar and the arrow). **(C)** Raster plots from the same MLI for spikes recorded in consecutive runs before, during and after photostimulation lasting either 10 ms (upper panel) or 100 ms (lower panel) at the time indicated by the blue boxes. **(D)** Pooled data for the spike frequency obtained in seven MLIs in control conditions and during 1 s photostimulation. Colored dots represent individual somata and black squares the corresponding average; error bars are SEM; *p* value for paired Student’s *t*-test: 0.003. **p ≤ 0.01. The power out of the fiber for all recordings shown was 6 mW.

**Figure 6:**
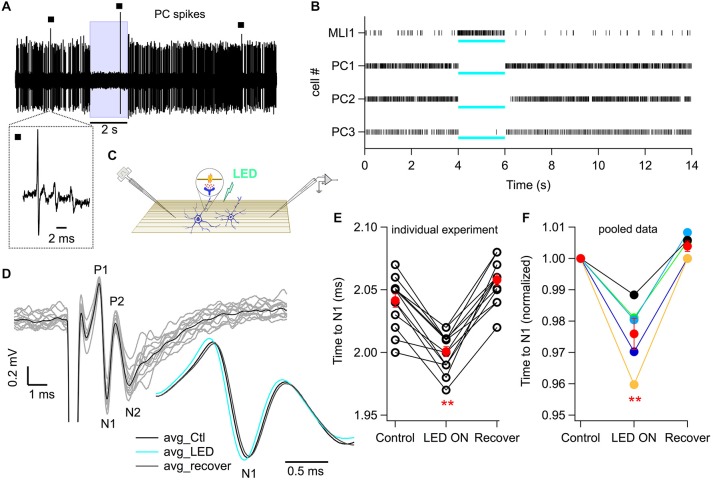
**Photostimulation of MLIs increases PF excitability *in vivo*. (A)** Representative trace of a continuous recording of Purkinje cell (PC) spikes from an anesthetized mouse in which MLIs express ChR2-YFP under the control of the nNOS promoter. Wide-field photostimulation with a 470 nm LED coupled to a 1 mm optical fiber (8 mW out of the fiber) placed in the solution bathing the craniotomy silences the simple spikes of PCs. The blue box marks the time of photostimulation. Black squares indicate complex spikes, identified by their multicomponent wave shape, visible in the expanded trace shown below. **(B)** Raster plots from four different animals in which spikes were recorded either from an MLI (MLI1) or from PCs (PC1, PC2 and PC3). PC3 corresponds to the example shown in **(A)**. Note the light-evoked increase in spike rate for the MLI and the silencing for the three PCs. The blue bars below each raster indicate the time of photostimulation. **(C)** Schematic of PF volley recording and photostimulation *in vivo*. PFs are depicted in brown and MLIs in blue. The inset portrays the suggested MLI-to-PF GABAergic signaling. **(D)** PF volley traces recorded by an extracellular electrode placed superficially in the ML of an anesthetized ChR2-YFP mouse. PFs were stimulated at 1 Hz (100 μs pulses, −70 V) with a theta glass pipette (see “Materials and Methods” Section for details). The black trace is the average of 14 repetitions (single traces in gray). The inset shows an expanded view of the averaged N_1_ wave, representing the AP propagating along the PFs, in control conditions (black), during light (blue; 1 s-long), and after recovery (gray). **(E)** The effect of photostimulation on the time to the N_1_ wave for the experiment shown in **(D)**. Each data triplet represents a set of volley recordings in control conditions, followed by LED stimulation and recovery. Red dots: mean ± SEM across trials. *p* = 6 × 10^−7^, paired Student’s *t*-test, *n* = 14 trials. **(F)** Effect of photostimulation on the time to N_1_ wave for averaged values from five animals. Red dots: mean ± SEM across the five animals. *p* = 0.007, paired Student’s *t*-test. **p < 0.01.

If activation of GABA_A_Rs results in a depolarization of PFs, then the time required to reach AP threshold should be diminished upon extracellular stimulation of PFs. This should shorten the delay for an extracellularly recorded PF volley, as previously observed in cerebellar slices (Dellal et al., 2012). It is unknown whether specific MLI activation can produce such effect, either in slices or *in vivo*. To address this issue we used optogenetic stimulation of MLIs in a nNOS-ChR2 BAC mouse line that expresses the light-activated cation channel, ChR2, specifically in MLIs (Kim et al., 2014). In this mouse line, experiments in slices from adult mice showed that optical stimulation elicited a vigorous activation of MLIs (example in Figures 5A–C), yielding an average increase in firing rate from 10–34 Hz (Figure 5D). Next we recorded extracellularly from neurons of the molecular layer *in vivo* while photostimulating by using an optical fiber located inside the bath above the craniotomy (Figures 6A,B). Upon light stimulation, we found a near complete abolition of firing in several neurons identified as Purkinje cells because of the presence of multicomponent wave shapes characteristic of complex spikes (see inset in Figure 6A; see Heiney et al., 2014 for a similar example in the same mouse line) and a strong increase in AP firing in one recording showing no multicomponent signals, which we attribute to an MLI (from 6.4 to 48.5 Hz). This demonstrates our ability to specifically stimulate MLIs *in vivo* and is in accord with a recent study that used this mouse line to identify the role of the MLI-PC circuit in control of motor output (Heiney et al., 2014). Extracellular recording of the PF volley was then performed in combination with photostimulation of MLIs (Figure 6C) and the rise times of the so-called N_1_ wave peaks (Figure 6D; Eccles et al., 1966) before and during photostimulation were compared. The duration of light excitation was set at 0.8 s, a moderate value in view of the long-lived (over 5 s) concerted activation of MLI clusters observed during motor tasks in awake animals (Ozden et al., 2012). Group data from five animals indicated that the delay of the PF volley was significantly and reversibly decreased by photostimulation (mean decrease: 45 ± 9 μs ; *p* = 0.007; paired *t*-test; Figure 6F), in accord with the decrease in PF volley latency observed in slices following GABA uncaging (Dellal et al., 2012). In two of these experiments (one of these illustrated in Figures 6D,E), there was sufficient signal-to-noise to properly measure individual traces, and in both cases significant differences were also found for the times to N_1_ peak before and during photostimulation (respective *p* = 0.003 and 6 × 10^−7^). In these two experiments, 1 s photostimulation after addition of SR95531 yielded smaller effects with a mean decrease of delay of 38 ± 5 μs in control (29 sweeps) and 19 ± 7 μs in the blocker (21 sweeps; *p* = 0.04). These results indicate that MLI activity induces a significant activation of GABA_A_Rs in PFs and furthermore, that the resulting effect is excitatory.

### Action of GABA_A_Rs on Basket Terminals

**Figure 7:**
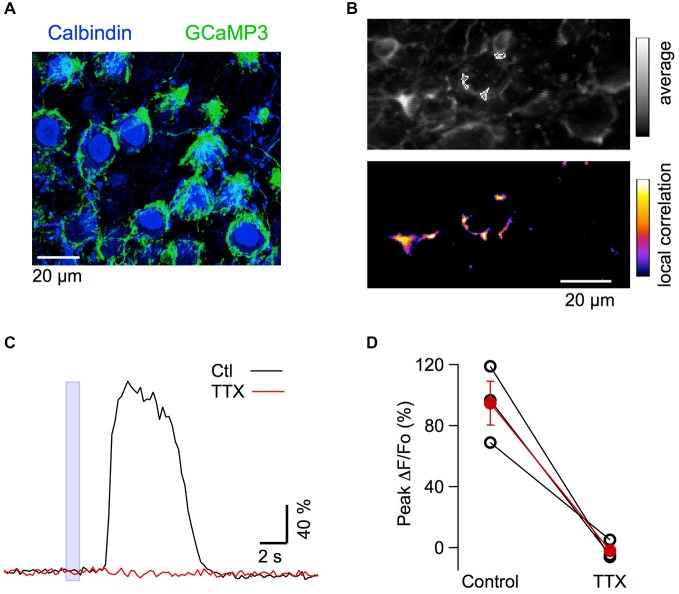
**MLI axonal responses to muscimol *in vivo*. (A)** Confocal z-stack projection (10 μm depth) from a transverse cerebellar slice of a mouse expressing GCaMP3. The slice was labeled with antibodies for calbindin (Cb). GCaMP3 expressing varicosities (green) surround the Cb positive PC somata (blue). **(B)** Top panel: average of 20 pre-stimulus two-photon images in the anesthetized mouse, at a depth of 150–200 μm. Lower panel: corresponding correlation image following muscimol application. The ROIs analyzed in **(C)** are drawn over the average image in **(B). (C)** Time course of the response to muscimol in control period and 24–34 min after addition of TTX to the solution bathing the craniotomy. Responses were averaged from all ROIs and over 3 consecutive trials in each condition. **(D)** Block by TTX of the fluorescent responses in basket varicosities. Pair plots for the peak ΔF/Fo values in control condition and in the presence of 2 μM TTX for three different animals; the red dots give mean ± SEM.

**Figure 8:**
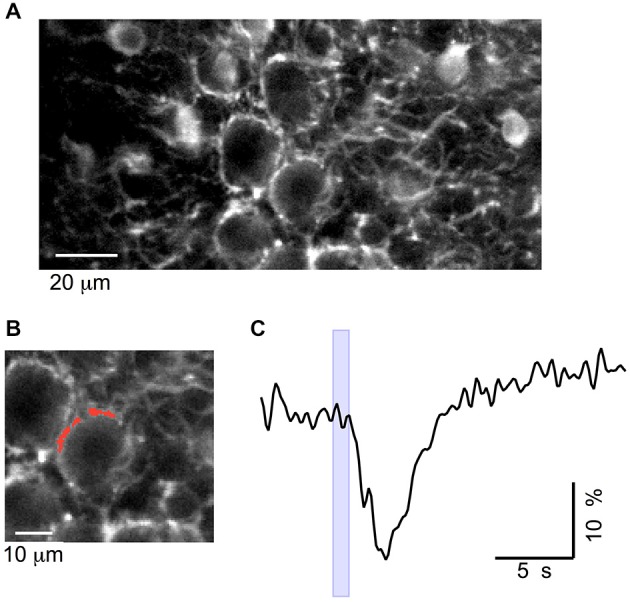
**Muscimol silencing of basket terminals *in vivo*. (A)** Two-photon image of the cerebellar cortex at the depth of 150–200 μm. **(B)** Zoom of the image in **(A)**, with the analyzed ROI depicted in red. **(C)** Time course of the change in fluorescence elicited by muscimol, at the time indicated by the blue box, with the iontophoresis pipette placed in front of the terminal.

Finally, we asked whether the excitation of PFs by GABA increases excitability at the output of the MLIs, thus yielding a positive feedback loop mediated by GABA. To investigate this possibility, we imaged GCaMP3 fluorescence in neurites surrounding PC somata, which can be unambiguously identified as basket cell terminals because of their characteristic “basket” morphology (Figure 7A). In eight animals, iontophoresis of muscimol, with the pipette placed 10–15 μm above the plane of PC somata, evoked Ca_i_ rises in basket terminals with peak values of 155 ± 25% (*n* = 14 spots; example in Figure 7B). In three of these experiments tetrodotoxin (TTX) was applied and abolished the muscimol-induced Ca_i_ rises in the terminals (Figures 7B–D; 101 ± 4% amplitude reduction). This finding is consistent with observations on MLI somata (Figure 4) and suggests that the effect is mediated by an excitatory action of muscimol on PFs. It has been reported that GABA_A_Rs are present in adult basket axons (Chan-Palay and Palay, 1978), and their direct activation could mediate excitatory responses. We therefore tested muscimol iontophoresis with the pipette placed in front of these terminals. Under these conditions, the responses were always inhibitory with an average ΔF/Fo value of −19 ± 2% (six terminals from five animals; Figure 8), ruling out the possibility of a direct excitatory effect of muscimol on MLI axons.

We conclude from these experiments that an elevation in bulk GABA concentration can induce a significant Ca_i_ rise in basket terminals, supporting the possibility that GABA release from such terminals participates in a positive feedback GABA loop.

## Discussion

### Excitatory GABA Loop

**Figure 9:**
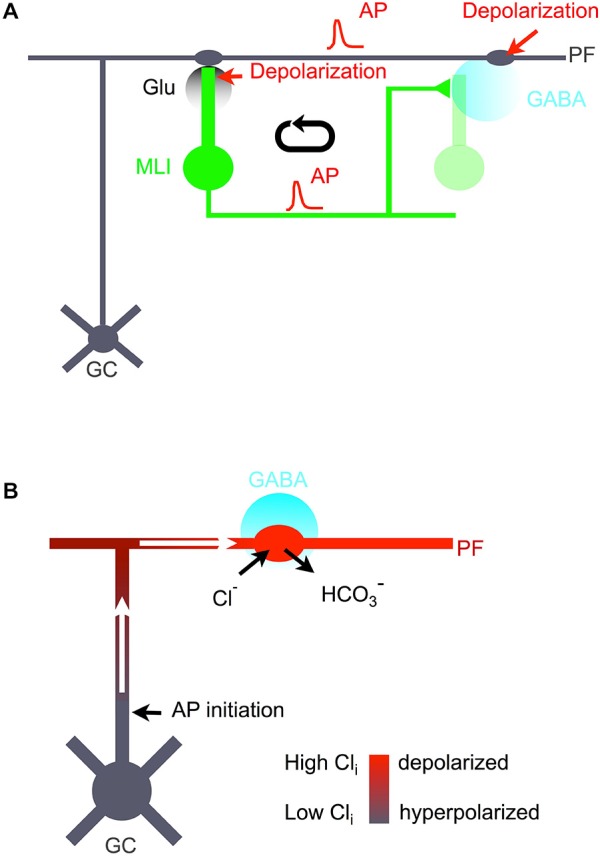
**Excitatory GABA loop. (A)** Cellular mechanism and functional role of excitatory GABA action in the cerebellar molecular layer. Following firing of granule cells (GC) and associated PFs (gray), postsynaptic MLIs (green) are depolarized, leading to AP firing and to increased Ca_i_ levels both in somata and in synaptic terminals. Synaptic terminal excitation produces GABA release (cyan). While released GABA affects postsynaptic cells (light green), spillover GABA diffuses to PF varicosities. These varicosities bear GABA_A_Rs and are depolarized by spillover GABA. This enhances AP firing in PFs, thus closing the loop. **(B)** Proposed model of GC excitation following activation of PF GABA_A_Rs. In PF varicosities, because of a favorable surface to volume ratio, activation of GABA_A_Rs leads to Cl^−^ entry, bicarbonate exit, and membrane depolarization (depicted in red). No AP is initiated at the site of GABA action because of shunting inhibition. However, the depolarized varicosity acts as a current sink, drawing depolarizing current flow along the axon cable (white arrows). This leads to AP initiation at sites where GABA_A_Rs are not activated, e.g., at the GC axon initial segment.

The principal result from our study is that PF excitability is enhanced *in vivo* by a positive feedback loop involving activation of GABA_A_Rs. While previous work in slices suggested this positive feedback loop (Stell et al., 2007; Pugh and Jahr, 2011; Stell, 2011; Dellal et al., 2012), the present work indicates that the various elements that have previously been studied in the slice environment operate *in vivo*. Our results suggest a circular chain of events inside a PF beam involving PF depolarization, PF firing, glutamate release, synaptic excitation of MLIs, GABA release by MLIs, and again GABA-induced PF depolarization (Figure 9A). Using different manipulations (extracellular stimulation of PF beam, iontophoretic application of muscimol, and optogenetic stimulation of MLIs), we could enter the GABA loop at different levels and in each case engage the entire mechanism. While these stimulations are clearly invasive, they result in patterns of activation that are not unlike those observed under physiological conditions. Thus we show that the GABA loop can operate when GABA release by MLIs is engaged by beam-like activation of PFs, an activity pattern recently shown to occur during forelimb stimulation (Cramer et al., 2013). Furthermore, from our single unit recordings in slices and *in vivo*, we estimate that the firing rate during photoactivation in the present work reaches values on the order of 40–50 Hz. This is within the firing rates recently reported from single unit recordings in the vermis of awake mice performing locomotion episodes lasting several seconds (Ozden et al., 2012).

MLIs are thought to participate in the cerebellar control of specific motor tasks (for review, see Jörntell et al., 2010). Their function has been highlighted by electrophysiological and behavioral studies of mice with mutations of GABA_A_Rs. These studies revealed abnormalities in the consolidation of vestibulo-ocular reflex learning (Wulff et al., 2009) as well as deficient motor coordination (Vinueza Veloz et al., 2014) when the inhibition between MLIs and PCs is disrupted. By stabilizing electrical activity in one PF/MLI/PC module, the GABA loop likely contributes to these functions.

Morphological data from P18–23 rats (Rieubland et al., 2014) suggests that a large proportion of the GABA release by MLIs occurs close to their somata, due to a higher density of axon collaterals in the proximal part of the axon. This indicates that upon beam PF excitation, spillover GABA will occur mainly inside the beam. Altogether, a plausible hypothesis is that MLI activity not only enhances PF firing inside the beam, via GABA spillover, but also inhibits MLI firing on the edge of the beam (by inhibitory MLI-MLI interactions), thus lowering the probability of PF firing at the edge of the beam. Thus, the proposed excitatory GABA loop is predicted to enhance PF firing in the PF beam and not in the surrounding volume; this should stabilize beam PF activity and sharpen spatial contrast within the molecular layer.

At the next stage of synaptic integration, in PCs, the inhibitory action of MLI-PC synapses restricts the parasagittal spread of signals during PF bursts in a frequency-dependent manner (Santamaria et al., 2007; Mapelli et al., 2010). Thus MLIs have the capability to either enhance or restrict the parasagittal spread of signaling by acting on PFs and on PCs respectively. The final outcome is likely to vary accordingly to the frequency and duration of the PF bursts. While the present work suggests that sustained MLI activity, as observed during mice performing locomotion (Ozden et al., 2012), leads to GABA loop activation, the possibility remains that for short PF bursts, as observed during sensory processing (Chadderton et al., 2004) the GABA loop would be weaker, potentially leaving the inhibitory action of GABA predominant.

### Cellular Mechanisms Underlying Excitatory Effects of GABA_A_Rs *in Vivo*

Phasic excitatory effects of GABA_A_Rs are ultimately linked to a high Cl_i_ at the location of GABA action. Several mechanisms have been proposed that can lead to elevated Cl_i_ levels, as follows: (i) First, in neonatal preparations, the balance between Na-K-Cl cotransporter (NKCC1)-associated accumulation of Cl^−^ and KCC2-associated extrusion of Cl^−^ is assumed to lead to depolarizing GABA action (review; Ben-Ari, 2002; but see Bregestovski and Bernard, 2012; Dzhala et al., 2012; Glykys et al., 2014). This mechanism is obviously excluded since our experiments were done in adult animals; (ii) A second proposal is gradual Cl^−^ accumulation in the somatodendritic compartment due to intense and prolonged receptor activation (Alger and Nicoll, 1979; Thompson and Gähwiler, 1989; Staley et al., 1995; Chavas et al., 2004). However, this mechanism has proven unable to lead to AP firing, presumably because massive GABA_A_R activation leads to shunting inhibition. Direct activation of GABA_A_Rs in MLIs by prolonged (several seconds long) muscimol stimulation can lead to Ca_i_ elevation due to Cl^−^ accumulation (Chavas et al., 2004), but this effect is unaffected by TTX, whereas in the present work, the excitatory responses to muscimol were abolished by TTX. The block of muscimol-induced MLI excitation by glutamate receptor antagonists (Figure 4) is a further argument against this second mechanism; and (iii) A third proposal has been that high Cl_i_ values occur in the axon, due to differential Cl_i_ homeostatic controls in the somatodendritic and axonal compartments (Price and Trussell, 2006; Szabadics et al., 2006).

Even though none of the above mechanisms applies directly to the granule cell-PF system, two lines of evidence support the possibility of a high Cl_i_ in GC and consequently in PFs. First, NKCC1 is enriched in adult GC (Seja et al., 2012) and references within) providing a potential mechanism for high Cl_i_ levels. A second factor enhancing Cl_i_ is tonic GABA action on GC (review, Farrant and Nusser, 2005), which *in vivo* activates high affinity extrasynaptic GABA_A_Rs (Chadderton et al., 2004; Duguid et al., 2012). In slices, blocking GABA_A_Rs decreases Cl_i_ in both GC and PFs, an effect reduced by genetic deletion of Best1 channels in glial cells, so that it has been proposed that tonic GABA released by glial cells maintains high Cl_i_ in GC and PFs (Lee et al., 2010). Recent Cl_i_ imaging work also shows that the high Cl_i_ occurs in PFs *in vivo*, due to tonic activation of GABA_A_Rs, and that this can render subsequent phasic GABA_A_Rs activation excitatory (K. Berglund, L. Wen, R. L. Dunbar, G. Feng, G. J. Augustine, under review). In addition to this glial effect, spillover GABA at the inhibitory synapses between Golgi cells and GC (Rossi and Hamann, 1998) will also contribute to elevating basal Cl_i_ in GC because of the diffusional constraints set by the glomerular structure (see Mapelli et al., 2014 for a recent review and references within).

As an alternative or as a complement to mechanism (iii), spillover GABA may lead to a Cl_i_ rise in PF varicosities by a bicarbonate-driven mechanism (Staley et al., 1995). Previous results in slices showed APs in granule cell somata following stimulation of axonal GABA_A_Rs (Pugh and Jahr, 2011), but underlying mechanisms have remained unclear. We propose that GABA_A_R activation leads to a rapid Cl_i_ accumulation in the varicosity due to the very high surface to volume ratio of these structures (see Brenowitz and Regehr, 2007 which reports an estimated mean of 0.63 ± 0.04 femtoliters for the volume of PF varicosities). This leads to local depolarization since the local potential follows the evolution of E_Cl_ (Staley et al., 1995). Once this depolarization is established the stimulated varicosities act as a current sink, generating a depolarizing current flow inside the axon cable (Figure 9B). PF axons have a very long cable length constant (Pugh and Jahr, 2013), so that a depolarization arising hundreds of microns away from the soma can reach in this manner the axon initial segment, initiating an AP (Figure 9B). While inside the GABA spillover zone, AP firing is inhibited by GABA_A_R-induced shunting, together with Na^+^ channel inactivation and K^+^ channel activation (Jackson and Zhang, 1995), distant AP initiation sites (e.g., at the axon initial segment) are not exposed to GABA and are therefore not shunted, allowing AP firing (Figure 9B). In retrospect, the reasons why GABA-induced Cl_i_ accumulation [mechanism (ii)] does not lead to AP firing in the somatodendritic compartment are presumably that the accumulation of Cl_i_ is limited by an unfavorable surface to volume ratio, and that AP firing is prevented by shunting inhibition. The situation is different in axons, so that a modification of mechanism (ii) linked to activation of axonal receptors is a plausible explanation for the excitatory effects of GABA described here. This modified mechanism, as illustrated in Figure 9B, is consistent with the present experimental finding that a comparatively large muscimol concentration is needed to obtain PF firing.

In summary, our study argues for excitatory effects of axonal GABA_A_Rs not only *in vitro*, but also *in vivo*. More generally, it supports the view that excitatory GABA actions occur in the adult mammalian brain and can regulate the cerebellar circuit under physiological conditions.

## Author Contributions

*In vivo* experiments and analysis: GA, JB. *In vitro* experiments: IL. Development of stereotaxic injections: JB. Development of *in vivo* imaging set-up: RF. Immunocytochemistry: AJ. ChR2 transgenic mouse line: GJA. Overall experimental design: IL, AM, GA. IL and AM wrote the paper and all authors participated in the final version of the manuscript.

## Conflict of Interest Statement

The authors declare that the research was conducted in the absence of any commercial or financial relationships that could be construed as a potential conflict of interest.
